# Correlation between higher-order aberrations and visual acuity recovery (CoHORT) after spectacles treatment for pediatric refractive amblyopia: A pilot study using iDesign measurement

**DOI:** 10.1371/journal.pone.0228922

**Published:** 2020-02-14

**Authors:** Chun-Fu Liu, Chung-Hsin Tseng, Chung-Ying Huang, Chi-Chin Sun, Meng-Ling Yang, Wei-Yi Chen, Ling Yeung

**Affiliations:** 1 Program in Molecular Medicine, National Yang Ming University, Taipei, Taiwan; 2 Department of Ophthalmology, Chang Gung Memorial Hospital, Keelung, Taiwan; 3 College of Medicine, Chang Gung University, Taoyuan, Taiwan; 4 Department of Ophthalmology, Chang Gung Memorial Hospital, Linckou, Taiwan; 5 Institute of Biochemistry and Molecular Biology, National Yang-Ming University, Taipei, Taiwan; National Yang-Ming University Hospital, TAIWAN

## Abstract

**Purpose:**

To determine the correlation between higher-order aberrations (HOAs) and best-corrected visual acuity (BCVA) recovery speed after spectacles treatment using iDesign measurements in refractive amblyopic children.

**Methods:**

This is a prospective case series. Children aged from 3 to 7 years with refractive amblyopia (Landolt C equivalent < 0.8) were recruited. All participants were followed for at least 6 months after full correction of the refraction error by spectacles. The HOAs were measured using iDesign before and after cycloplegia at first visit and at 3-month intervals. Then correlation between BCVA recovery after treatment for 6 months and HOAs was determined.

**Results:**

We analyzed 24 eyes of 12 children (mean age, 4.5 years). Baseline mean BCVA was logarithm of minimal angle of resolution (logMAR) 0.335 (Landolt C equivalent 0.46), which improved to logMAR 0.193 (Landolt C equivalent 0.64) after treatment with full-correction spectacles for 6 months. The amblyopic eye BCVA recovery was negatively correlated with tetrafoil with/without cycloplegia (*P* = 0.006 and 0.022, respectively) and trefoil with cycloplegia (*P* = 0.049).

**Conclusions:**

trefoil and tetrafoil measured with iDesign negatively correlates with the BCVA recovery speed of refractive amblyopic eyes after spectacles treatment in this pilot study. The current study results may aid in further investigation for diagnosis and treatment of refractory refractive and idiopathic amblyopia.

## Introduction

Amblyopia, a common ophthalmologic disease with a prevalence of 1.6%–3.6%, is considered to be due to insufficient foveal stimulation during a critical period. It has three main types: refractive, strabismic and deprivation amblyopia[1–4], of which, refractive amblyopia is the most common[5, 6].

Higher-order aberrations (HOAs) may be a critical cause of monocular idiopathic amblyopia[7, 8]. Vincent et al. also indicated the HOAs is significantly different between an amblyopic eye and a normal fellow eye[9]. However, most studies have had a small sample size and measured the HOAs using machines with older technology; the results varied in different studies from different materials and methods, and are still not conclusive[1, 7–13]. A recent novel study using HOAs-correcting, real-time, closed-loop adaptive optics perceptual learning system demonstrated that best-corrected visual acuity (BCVA) and contrast sensitivity significantly improved in adolescents’ refractive amblyopic eyes (mean age, 16 years)[14]. Thus, HOAs may influence the BCVA recovery of refractive amblyopia after spectacles treatment.

A higher-order aberrometer can be used in refractive surgery to improve postoperative visual quality[15, 16]. iDesign (Johnson & Johnson Vision, Santa Ana, CA, USA) is a new-generation aberrometer designed based on Hartmann–Shack theory to eliminate HOAs after refractive surgery and improve final visual quality. Surgery using iDesign is referred to as wavefront-guided refractive surgery and has provided satisfactory results in many patients, so HOAs measured by iDesign may be crucial in determining visual quality[15–21]. Moreover, iDesign improves accuracy and resolution of HOAs measurements since it has more detection points and greater resolution than other aberrometers[16].

To our knowledge, no cohort study thus far has measured HOAs by using iDesign in pediatric amblyopic eyes and we hypothesized that HOAs measured by iDesign may influence BCVA recovery speed of amblyopic eyes after spectacles treatment. Therefore, this prospective study determines the correlation between BCVA recovery and HOAs measured using iDesign in refractive amblyopic eyes after full lower-order aberrations correction by spectacles for 6 months during a critical period.

## Materials and methods

### Subjects

This prospective, single-center, longitudinal study was conducted from August 2015 to December 2017 at Chang Gung Memorial Hospital, Taipei, Taiwan. Children aged from 3 to 7 years, with refractive amblyopia (Landolt C equivalent < 0.8) in both eyes who cooperated with iDesign and BCVA measurements were recruited.

This study was approved by the ethical and scientific committee of the Institutional Review Board of Chang Gung Memorial Hospital (approval no.: 103-4554B) and was performed in accordance with the tenets of the Declaration of Helsinki. We received the formal consent to participate and consent for publication from the parents or legal guardians of all participants in the study after thorough explanation of the study protocol.

### Ocular examination protocol

All children received complete, standard, scheduled ocular examinations at the first visit and at regular trimonthly follow-ups. Uncorrected distance vision acuity and BCVA measurements, slit-lamp anterior segment examination, and measurements of objective refraction errors before and after cycloplegia by using an autorefractometer (Auto Ref/Keratometer-1a/ARK-1; Nidek Co., Ltd., Aichi, Japan) were performed at every visit. HOAs measurements before and after cycloplegia were also performed using iDesign at the same visit. Moreover, axial length measurements (IOLMaster 500, Carl Zeiss Meditec AG, Jena, Germany) were performed at first visit, every 6 months and as necessary. Fundus photography and macular optical coherence tomography (Optovue RTVue-100; Optovue Inc., Fremont, CA, USA) scan were also performed at baseline and thereafter annually.

For cycloplegia induction, phenylephrine (10%) and tropicamide (1%) were administered together every 10 minutes for three times in each eye; after waiting at least 1 hour of the first administration. Pupil enlargement with no light reflex was used for confirmation before examinations requiring cycloplegia.

### HOAs measurements

**Fig 1:**
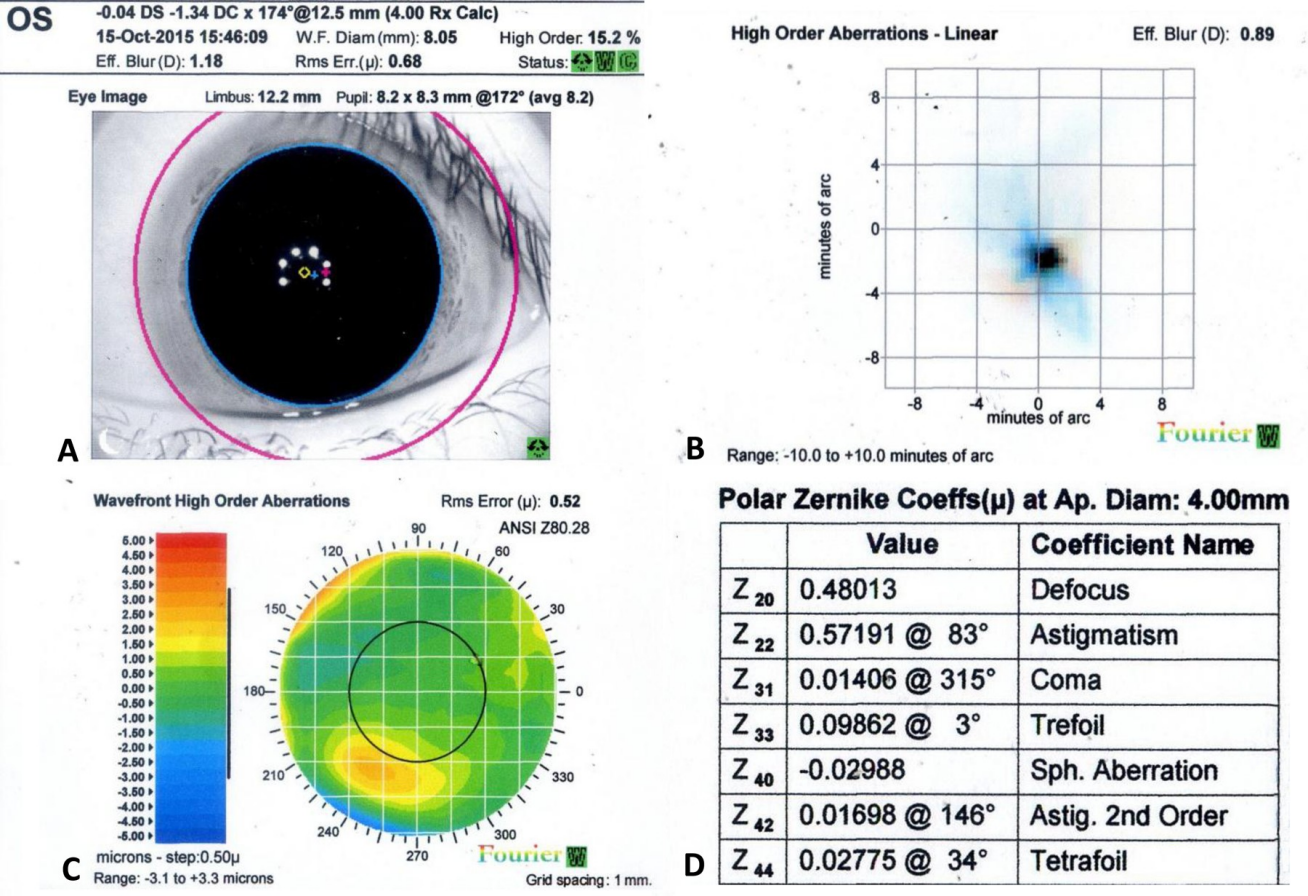
Example to explain data collection: Left eye of a 5-year-old girl who underwent iDesign measurements after cycloplegia. (A) Eye image and refraction error power (diopter), wavefront reconstruction based on Zernike polynomials for all-order aberrations root mean square (RMS) error (μ), higher-order aberrations (HOAs) rate (%), all-order aberrations effective blur (diopter), and quality check signal light; (B) Point spread function and HOAs effective blur (diopter) using Fourier reconstruction; (C) HOAs Wavefront error map and HOAs RMS error (μ) using Fourier Reconstruction; (D) Zernike table and Polar Zernike coefficients (μ) at aperture diameter of 4 mm.

HOAs data acquired using a 4-mm pupil diameter calculation setting for iDesign were statistically analyzed. Fig 1 shows a sample eye of measurement report obtained from iDesign.

The patients were asked to blink once just before the scan and focus on the fixation target. The measurements were repeated five times for each eye. The best measurement was selected by an ophthalmologist (C.F. Liu) experienced with wavefront-guided refractive surgery according to the manufacturer’s suggestions, such as larger pupil size, better wavefront quality-check signal light, and closer refraction error to objective cycloplegic refractive error. The average of all acquired data for the same eye were used for further analysis.

### Amblyopic eye treatment protocol

All patients received full correction with spectacles prescribed as cycloplegic refraction error by using the autorefractometer measurements as a standard treatment[22]. Treatment was initiated after successful measurement of both BCVA and cycloplegic refraction. BCVA recovery speed was defined as BCVA improvement after 6 months treatment with spectacles with good compliance [calculated as BCVA 6 months after treatment minus baseline BCVA (logarithm of minimal angle of resolution, logMAR)]. We also planned to replace the spectacles when differences in the refraction errors and spectacle lens powers was >0.50 diopters of spherical or astigmatism power (axial length change wound be used to verify spherical power change before changing prescription)[23]. At every visit, we educated the parents to ensure full time wearing of the spectacles and ensure compliance.

#### Exclusion criteria

The exclusion criteria were as follows: (1) follow-up duration of <6 months after initiating treatment, (2) poor quality of HOAs data even after repeated measurements, (3) the better eye had once received occlusion or pharmacologic penalization therapy for amblyopia treatment during follow-up, (4) patient refused full correction of the spherical or astigmatism refraction error, (5) average spectacle-wearing time less than 90% of waking hours (information was acquired carefully from the parents at every visit), (6) presence of any form of strabismus, (7) use of cycloplegic agent for myopia control during follow up period, (8) any visible structural defect on optical coherence tomography or fundus photography examination.

#### Statistical analysis

Correlation coefficients between trefoil or tetrafoil and visual acuity recovery speed were assumed to be 0.6. Given alpha level of 5% and power of 85%, the required eyes were 21.

Data were analyzed using SPSS (version 20.0; SPSS, Inc., Chicago, IL, USA). Correlation between BCVA recovery speed and average HOAs of all successful measurements for each eye was determined using the Generalized Estimating Equations (GEE) model. Regarding the two eyes analysis of one patient, the GEE model was also used to account for the outcome dependency among two eyes of one case. The linking function was identity and distribution was normal in the GEE. Exchangeable working correlation and robust standard error were adopted to obtain the significance of parameters. Statistically significant differences were defined using a two-tailed *P* of <0.05.

## Results

In total, 56 eyes of 28 cases were recruited, of which, 16 eyes (8 cases), 4 eyes (2 cases), 6 eyes (3 cases), and 6 eyes (3 cases) were excluded because of loss to follow-up after baseline examination, strabismus, only poor quality of HOAs examination data even after repeated measurements, and cover therapy during the follow-up period, respectively. Finally, 24 eyes of 12 cases were analysed. Table 1 summarizes the clinical characteristics and objective refraction errors of all studied subjects with cycloplegia, the mean age was 4.5 years (range, 3 to 6), the spherical power was +1.36 ± 1.92 diopter (D) (mean ± standard deviation, range, -2.0 to +4.75 D); the astigmatism power was -2.78 ± 1.28 D (mean ± standard deviation, range, -0.75 –-5.00 D). Table 2 summarizes the treatment and HOAs data, it is also notable that no subject received prescription change for spectacles during follow up.

**Table 1:** Baseline characteristics and objective refraction error of studied subjects with cycloplegia.

Subjects No.	Age	Gender	Refraction Error			
			OD		OS	
			Sphere	Cylinder	Sphere	Cylinder
**1**	5	F	0.75	-1.75	0.75	-1.75
**2**	4	F	0.50	-2.00	0.25	-2.00
**3**	5	M	0.75	-1.75	0.75	-1.75
**4**	5	M	0.50	-3.50	0.00	-2.00
**5**	6	M	4.75	-3.50	4.50	-3.25
**6**	5	M	1.75	-4.75	1.75	-4.75
**7**	5	M	1.00	-2.50	-0.25	-5.00
**8**	3	F	4.50	-4.00	3.75	-2.00
**9**	5	M	-0.25	-2.25	-1.00	-2.00
**10**	3	F	3.75	-3.00	3.50	-2.25
**11**	4	F	2.00	-0.75	2.25	-1.00
**12**	4	F	-1.50	-4.75	-2.00	-4.50

F: female; M: male. Age unit is years of age.

**Table 2:** Treatment and higher-order aberrations data of studied patients.

**Parameters Mean ± SD**	**Cases (n = 24)**	
**BCVA baseline (LogMAR)**	0.335 ± 0.160	
**BCVA after 6 months treatment (LogMAR)**	0.193 ± 0.156	
**Follow-up period (months)**	10.3 ± 2.5	
**HOAs related parameters measured by iDesign**	**Without cycloplegia**	**With cycloplegia**
**Number of measurements per eye (times)**	4.1 ± 1.1	4.1 ± 1.0
**All-order aberrations RMS error (μ)**	1.86 ± 1.24	1.61 ± 1.00
**HOAs RMS error (μ)**	0.37 ± 0.21	0.53 ± 0.21
**HOAs (%)**	8.2 ± 5.1	8.4 ± 4.0
**All-order aberrations effective blur (D)**	3.21 ± 2.14	2.93 ± 1.59
**HOAs effective blur (D)**	0.77 ± 0.51	0.92 ± 0.36
**Coma (μ)**	0.0669 ± 0.0421	0.0674 ± 0.0459
**Trefoil (μ)**	0.0577 ± 0.0227	0.0702 ± 0.0375
**Sphere aberration (μ)**	0.0026 ± 0.0248	-0.0026 ± 0.0246
**Tetrafoil (μ)**	0.2713 ± 0.0170	0.0221 ± 0.0117

BCVA: best-corrected visual acuity; D: diopter; HOAs: higher-order aberrations; LogMAR: logarithm of minimal angle of resolution; RMS: root mean square; SD: standard deviation.

Table 3 summarizes the statistical results of the GEE model showing that BCVA improvement negatively correlates with the tetrafoil without cycloplegia after treatment for 6 months, significantly (*P = 0*.*006*). In addition, when treating BCVA 6 months after treatment minus baseline BCVA as the dependent variable, the result showed BCVA baseline is also negatively correlated to BCVA improvement (LogMAR) (unadjusted B = -0.599, CI = -0.787 ~ -0.411, *P<0*.*001*). It is also notable that the observed effect of tetrafoil remains significant after adjusting for the BCVA baseline (adjusted B = 5.407, CI = 2.387 ~ 8,427, *P<0*.*001*; data not shown).

**Table 3:** Correlation of BCVA recovery with age, and higher-order aberrations data without cycloplegia after treatment for 6 months.

Parameter	Univariate analysis			
	B	95% CI		*P* value
		Lower	Upper	
**Age (years)**	-0.052	-0.129	0.025	0.187
**All-order aberrations RMS error (μ**)	0.008	-0.032	0.048	0.694
**HOAs RMS error (μ**)	0.035	-0.193	0.263	0.763
**HOAs (%)**	-0.006	-0.019	0.007	0.371
**All-order aberrations effective blur (D)**	0.005	-0.018	0.028	0.658
**HOAs effective blur (D)**	0.057	-0.029	0.143	0.194
**Coma (μ**)	0.099	-1.158	1.356	0.877
**Trefoil (μ**)	-0.837	-2.453	0.779	0.310
**Sphere aberration (μ**)	0.603	-0.547	1.753	0.304
**Tetrafoil (μ**)	5.146	1.472	8.820	0.006*

Dependent variable: BCVA 6 month after treatment minus baseline BCVA.

* *P* <0.05, generalized estimating equations.

BCVA: best-corrected visual acuity; D: diopter; HOA: higher-order aberrations; RMS: root mean square.

Table 4 summarizes the statistical results of the GEE model with cycloplegia, showing that tetrafoil and trefoil both negatively and significantly correlate with BCVA improvement (*P* = 0.022 and *P* = 0.049, respectively). However, tetrafoil showed higher correlation after adjustment, although the difference is small (*P* = 0.008 versus *P* = 0.052 after adjustment). Since the BCVA baseline is also negatively correlated to BCVA improvement (LogMAR), it is notable that the observed effect of tetrafoil and trefoil is not significant after adjusting for the BCVA baseline (data not shown).

**Table 4:** Correlation of BCVA recovery with age, and higher-order aberrations data with cycloplegia after treatment for 6 months.

Parameters	Univariate analysis				Multivariable analysis			
	B	95% CI		*P* value	B	95% CI		*P* value
		Lower	Upper			Lower	Upper	
**Age (years)**	-0.052	-0.129	0.025	0.187				
**All-order aberrations RMS error (μ**)	0.030	-0.002	0.063	0.070				
**HOAs RMS error (μ**)	0.003	-0.243	0.250	0.979				
**HOAs (%)**	-0.008	-0.022	0.006	0.260				
**All-order aberrations effective blur (D)**	0.019	-0.003	0.042	0.090				
**HOAs effective blur (D)**	0.004	-0.141	0.149	0.960				
**Coma (μ**)	0.471	-0.597	1.540	0.387				
**Trefoil (μ**)	1.296	0.002	2.590	0.049*	1.307	-0.013	2.627	0.052
**Sphere aberration (μ**)	0.092	-0.899	1.083	0.856				
**Tetrafoil (μ**)	2.834	0.413	5.254	0.022*	2.871	0.751	4.991	0.008*

Dependent variable: BCVA 6 month after treatment minus baseline BCVA.

* *P* <0.05, generalized estimating equations.

BCVA: best-corrected visual acuity; D: diopter; HOAs: higher-order aberrations; RMS: root mean square.

## Discussion

To the best of our knowledge, this is the first study exploring the correlation between BCVA recovery and iDesign-measured HOAs in refractive amblyopic eyes after spectacles treatment for 6 months during a critical period. It is also the first study providing iDesign exam data for preschool-aged (3 to 6-year-old) children. We found that the BCVA recovery speed negatively correlated with tetrafoil with/without cycloplegia and trefoil with cycloplegia after treatment. However, tetrafoil has higher correlation when considering both of them. Thus, HOAs measured by iDesign may provide BCVA recovery information for refractive amblyopic eyes after spectacles treatment.

Trefoil and tetrafoil have the highest correlation on BCVA recovery after spectacles treatment in the current study; consistent with previous studies that have shown image quality disturbance by the HOAs may lead to idiopathic amblyopia[7, 8]. Prakash et al. determined the maximum differences of R-squared variability estimation value (stepwise regression analysis model in one Zernike polynomial) in the trefoil, coma, and tetrafoil between idiopathic amblyopic eye and normal fellow eye[8]. However, some studies had differing results probably because of different study designs in materials and methods[1, 9, 10, 12, 13]. Nevertheless, HOAs affects the image quality theoretically and clinically, so HOAs, particularly the trefoil and tetrafoil measured using iDesign, may influence BCVA recovery after spectacles treatment of refractive amblyopic eye. Additional large-scale studies confirming this observation are warranted.

In our patients, initial BCVA was not very poor, at least over 0.2 with only refractive problem (average 0.4 in Landolt C chart), so we prescribed full-correction spectacles as the first-line treatment and then followed up at 3-month intervals[22]. Through this we determined HOAs by iDesign and used the average HOAs measurement of each visit for each eye for analysis to improve reliability. We also excluded patients receiving occlusion or pharmacologic penalization therapy or cycloplegic agent for myopia to prevent their influences. Therefore, patients with lower-order aberrations fully corrected by spectacles with only HOAs left in daily life were included to suit the study purpose. However, the patients enrolled in the study receiving regular exams tended to have better compliance for wearing spectacles, so the BCVA recovery speed may be overestimated. But this would further contribute to more reliable results due to greater compliance with spectacles treatment in the current study.

Most studies have used diagnosis-based aberrometers for HOAs measurements, such as the Complete Ophthalmic Analysis System (COAS Wavefront Sciences Inc, Albuquerque, NM, USA), KR-1w (Topcon Co., Tokyo, Japan), or iTrace Visual Function Analyzer (Tracey Technologies, Houston, TX, USA). Others have used older, lower-resolution models with HOAs treatment function that are no longer available [e.g., WaveScan Wavefront System (AMO, Santa Ana, CA, USA) and Zywave II (Bausch & Lomb, Rochester, NY, USA)][1, 7–10, 12, 13]. The new-generation machines, such as iDesign, have both higher measurement resolution and shorter examination time, so they may provide more reliable HOAs data in children, who tend to move about. Furthermore, iDesign can be used for HOAs correction in combination with an excimer laser (VISX STAR S4; Johnson & Johnson Vision, Santa Ana, CA, USA). Thus, this combination may help develop customized HOAs-corrected contact lens treatment for refractory refractive or idiopathic amblyopia after further investigation.

Pediatric HOAs measurement should be evaluated after cycloplegia to prevent the effects of accommodation on HOAs changes[24]; however, manufacturer suggests measuring without cycloplegia for refractive surgery on adults. In this pilot study we performed measurements and analysis under both conditions for more detailed information. We also selected pupil diameter of 4 mm for HOAs analysis, regardless of cycloplegia, because it had the greatest influence on image quality[1, 7, 9, 10, 12, 13, 24–27]. The information providing in the current study would be useful for future large-scale studies design.

Since parents are highly concerned when their children are diagnosed with amblyopia, there is a need for a safe and reliable tool that can provide BCVA recovery information of amblyopic eye after spectacles treatment. The current pilot study shows that iDesign may be an available tool for this purpose in clinical practice. Of note is that BCVA baseline is also negatively correlated to BCVA improvement after spectacle treatment for 6 months, this may be explained by the fact that the worse initial BCVA may have more room for visual improvement. However, results of previous studies varied due to different designs addressing different types of amblyopia[28–30]. Among these issues, most subjects were anisometropic and had strabismic amblyopia, with little consideration for a correlation between BCVA baseline and BCVA improvement speed in bilateral refractive amblyopia. Thus, a further large scale prospective study addressing on this issue should be performed in the future [28, 31].

This study has some limitations: small sample size, lack of control group, high dropout rate and some data loss due to poor-cooperation of the children during follow-up examinations. The high dropout rate resulted from the long examination time every visit and the poor cooperation of children during continuous follow-up examinations, despite our best efforts. To counter the poor-cooperation of the children, we repeated the examination several times and encouraged the children to cooperate as much as possible. But despite its limitations, this pilot study provides information regarding the influence of HOAs on BCVA recovery of refractive amblyopic eye after spectacles treatment. It also provides information useful for future large-scale prospective studies.

In conclusion, the current short-term pilot study that measured HOAs using iDesign demonstrates that after spectacles treatment for 6 months, the BCVA recovery from refractive amblyopia was faster with more negative tetrafoil with/without cycloplegia and more negative trefoil with cycloplegia. Thus, iDesign HOAs measurement with/without cycloplegia may provide important clinical information regarding BCVA recovery from refractive amblyopia after spectacles treatment, though further large-scale research with longer follow-up time is necessary to confirm these findings. The current study results may also aid in further investigation for diagnosis and treatment of refractory refractive and idiopathic amblyopia.
